# Evaluation of Genotoxic and Hemolytic Effects of *Aphanizomenon flos-aquae* and *Microcystis aeruginosa* Biomass Extracts on Human Blood Cells In Vitro

**DOI:** 10.3390/microorganisms12112208

**Published:** 2024-10-31

**Authors:** Nevena B. Đorđević, Jovana Tubić Vukajlović, Olivera Milošević-Đorđević, Vladimir B. Mihailović, Nikola Z. Srećković, Aleksandra B. Rakonjac, Snežana B. Simić

**Affiliations:** 1 Department of Biology and Ecology, Faculty of Science, University of Kragujevac, Radoja Domanovića 12, 34000 Kragujevac, Serbia; jovana.tubic@pmf.kg.ac.rs (J.T.V.); olivera.djordjevic@pmf.kg.ac.rs (O.M.-Đ.); aleksandra.mitrovic@pmf.kg.ac.rs (A.B.R.); snezana.simic@pmf.kg.ac.rs (S.B.S.); 2 Department of Genetics, Faculty of Medical Sciences, University of Kragujevac, Svetozara Markovića 69, 34000 Kragujevac, Serbia; 3 Department of Chemistry, Faculty of Science, University of Kragujevac, Radoja Domanovića 12, 34000 Kragujevac, Serbia; vladimir.mihailovic@pmf.kg.ac.rs (V.B.M.); nikola.sreckovic@pmf.kg.ac.rs (N.Z.S.); 4 Institute for Vegetable Crops Smederevska Palanka, Karađorđeva 71, 11420 Smederevska Palanka, Serbia

**Keywords:** cyanobacteria, cylindrospermopsin, anatoxin-a, microcystin, DNA damage, reservoir

## Abstract

This study explores the in vitro effects of cyanotoxins from the methanolic extract of the cyanobacteria *Aphanizomenon flos-aquae* and *Microcystis aeruginosa* on human blood cells, with samples drawn from the Gruža reservoir in Serbia. These cyanobacteria, which made up 98.5% of the reservoir’s phytoplankton, reached densities of 4,656,450 cells mL^−1^, with *A. flos aquae* (3,105,120 cells mL^−1^) as the dominant species, followed by *M. aeruginosa* (1,480,130 cells mL^−1^). A cyanotoxin analysis of biomass detected anatoxin-a (3.56 µg g^−1^), cylindrospermopsin (6.86 µg g^−1^), microcystin LR (0.87 µg g^−1^), and microcystin RR (2.47 µg g^−1^). This study assessed the genotoxic potential of the methanolic extract of the cyanobacterial biomass by evaluating the DNA damage and the Genetic Damage Index (GDI) in peripheral blood lymphocytes (PBLs) from healthy donors. The results showed a dose-dependent increase in the DNA damage, from 35.67 ± 4.93% at 10 µg mL^−1^ to 95.67 ± 1.53% at 100 µg mL^−1^, with a corresponding rise in the GDI from 0.61 ± 0.02 to 2.39 ± 0.07. The extract also caused the concentration-dependent hemolysis of red blood cells, with 5.63% hemolysis at the highest concentration (200 µg mL^−1^). These findings underscore the significant genotoxic risks posed by cyanotoxins from biomass extracts of *A. flos aquae* and *M. aeruginosa*, particularly in water sources used for human consumption.

## 1. Introduction

Safe drinking water is crucial for human health, and access to clean water has been a key factor in the development and decline of civilizations. With the global population projected to reach 10 billion by the mid-century, pressure on water resources will intensify [1]. Research, management, and policy are crucial for addressing health risks from water use, particularly from cyanobacteria and their toxins [2]. Increasing cyanobacterial blooms, driven by eutrophication from human-induced phosphorus and nitrogen inputs, along with climate change, threaten global water quality [3,4,5,6,7,8]. Both globally and in Serbia, the rising frequency of cyanobacterial blooms is exacerbating health risks and underscoring the urgent need for effective management strategies [9,10,11]. Cyanobacteria possess the ability to produce a wide range of secondary metabolites, with well-characterized biosynthetic pathways for many compounds. The importance of monitoring these cyanobacterial populations and their associated toxins has been widely recognized by the World Health Organization (WHO) [1]. Given the health risks posed by cyanobacterial blooms, the WHO has established guidelines and recommended alert levels to safeguard public health, emphasizing the need for the regular surveillance of water sources used for human consumption. While guidelines exist for the acceptable cyanotoxin concentrations in drinking water, including microcystins (MCs) at 1 µg L⁻¹, cylindrospermopsin (CYN) at 3 µg L⁻¹, anatoxin-a (ATX) at 30 µg L⁻¹, and saxitoxin (STX) at 3 µg L⁻¹, the actual impact of these toxins on human health, especially at the cellular level, remains underexplored. Most research has focused on detecting toxins in water rather than studying their direct biological effects. Despite the recognized risks, systematic monitoring of cyanotoxins has not yet been implemented in many reservoirs. This highlights the critical need for both ongoing monitoring and further investigation into the biological effects of cyanotoxins [12]. Given the widespread prevalence of cyanobacteria compared to many anthropogenic water contaminants, cyanotoxins are likely to occur more frequently and at potentially concerning levels [13].

Cyanotoxins have been shown to have a broad spectrum of toxic effects, with experimental studies on animals revealing that these toxins can target various organs. Neurotoxins like anatoxin-a disrupt the nervous system, while hepatotoxins such as microcystins induce liver damage through oxidative stress. Other cyanotoxins can affect the kidneys, gastrointestinal system, and skin [14]. These findings from animal studies have been critical in setting health guidelines for cyanotoxin exposure, particularly in drinking water, where the safety of human populations is at stake [15].

Human exposure to cyanotoxins can occur through drinking contaminated water, recreational activities, or consuming fish and shellfish from affected water bodies. Acute exposure can cause gastrointestinal disturbances, skin irritation, and respiratory issues, while chronic exposure may lead to liver damage, neurotoxicity, or even tumor promotion [1]. Despite these risks, most studies to date have focused on the effects of purified cyanotoxins or laboratory-cultured cyanobacterial strains, neglecting the examination of whole cyanobacterial biomass directly sourced from natural environments [16,17]. This is a significant gap in research, as human exposure often involves contact with a complex mixture of cells, toxins, and metabolites present in natural cyanobacterial populations.

The field of cyanobacterial toxicology has advanced through the use of various bioassays that allow researchers to explore different aspects of cyanotoxin action [15,16,17,18]. However, the majority of these bioassays focus on individual components of cyanobacterial toxicity, often involving purified toxins, and do not fully capture the complex interactions that occur in natural cyanobacterial blooms. Additionally, in vivo animal studies remain valuable for risk assessment but come with ethical concerns, leading to an increased reliance on in vitro methods that can provide human-relevant data without the need for animal testing.

In many studies, cyanobacterial toxicity has been assessed using a variety of organisms and test systems, but there remains a limited understanding of how natural cyanobacterial biomass, rather than isolated toxins, affects human health. Furthermore, the use of human cells in bioassays is still relatively underexplored [19,20,21]. While in vitro studies are increasingly recognized for their potential to elucidate biochemical mechanisms of toxicity, few have examined the direct effects of cyanobacterial biomass on human cells, particularly blood cells, which are a key target for assessing both genotoxicity and hemolytic activity.

The WHO [1] emphasizes the need, not only to monitor cyanotoxin presence, but also to rigorously control cyanobacterial biomass, particularly in multipurpose reservoirs. Implementing an Alert Level Framework (ALF), as suggested by WHO [1], which includes visual assessments, microscopy, and toxin quantification, would provide a more thorough and responsive strategy for managing the risks associated with cyanotoxins in drinking water supplies.

In accordance with the recommendation of WHO, future research, in addition to focusing on refining and standardizing bioassays for cyanotoxicity, should particularly emphasize testing cyanobacterial biomass from natural populations, identifying new model organisms, and integrating emerging technologies such as in vitro models and computational approaches to minimize the use of animals in research [22,23,24].

As most reservoirs used for water supply are multi-purpose, there is significant potential for multiple exposures to cyanobacteria, especially during blooms, through activities such as irrigation, sports, and recreation. Therefore, understanding and managing the impact of toxic cyanobacteria biomass on human health is becoming increasingly critical within the broader context of global water safety [1].

The aim of this study was to investigate the in vitro impact of toxins from the cyanobacterial biomass extracts of *Aphanizomenon flos-aquae* Ralfs ex Bornet and Flahault 1886 and *Microcystis aeruginosa* (Kützing) Kützing 1846 from the water supply reservoir on human blood cells. By analyzing the genotoxic and hemolytic potential of this cyanobacterial biomass extract, this study aims to enhance our understanding of potential health risks, particularly in the context of human exposure through contaminated water sources.

## 2. Materials and Methods

### 2.1. Sampling

Water bloom biomass samples of *Aphanizomenon flos-aquae* and *Microcystis aeruginosa* were collected from the Gruža reservoir. The Gruža reservoir was created in 1983, by damming the middle part of the Gruža River [11]. The Gruža reservoir is 10 km long, with a width ranging from 0.2 km to 1.5 km, and a maximum volume of 64.6 × 10^6^ m³ [25]. Its maximum depth is 31 m, with an average depth of 6.5 m, and its water levels fluctuate between 3 and 5 m [25]. The primary purpose of the reservoir is to supply water to the City of Kragujevac. It also serves for industrial use, irrigation, sports and recreation, flood protection, sediment retention, and improving low water regimes downstream. The reservoir is mostly shallow, with the banks surrounded by agricultural fields and meadows, and five smaller tributaries flowing into it [11,26].

Sampling of the biomass was carried out in October 2023, from the shallowest part of the reservoir, where the biomass was most abundant. Phytoplankton sampling was performed in the same spot in accordance with the standard method [27] EN 16698:2016. Qualitative samples were taken by pulling the plankton net (mesh diameter 22 μm), while quantitative samples were taken using Ruttner’s bottle (2 L). A quantitative analysis of the phytoplankton was carried out using the Utermöhl method [28] with an inverted microscope (Motic AE 2000 Series, Hong Kong, China), and it was expressed as the number of cells per mL. The microscopic identification of *Aphanizomenon flos-aquae* and *Microcystis aeruginosa* was carried out on the basis of their morphological characteristics as seen using a microscope (Motic BA310, Nikon Eclipse E100, Hong Kong, China) with magnification up to 40–100×. Identification was carried out using the following literature [29,30]. Phytoplankton analysis was conducted in the Hydrobiology Laboratory of the Department of Biology and Ecology at the Faculty of Science, University of Kragujevac.

### 2.2. Extraction Procedure

Cyanotoxin extraction for cyanotoxin analysis was performed according to the World Health Organization (WHO) protocol [1,31]. The cyanobacteria biomass, containing both *Aphanizomenon flos-aquae* and *Microcystis aeruginosa*, was removed from the water sample via centrifugation at 2500× *g* for 15 minutes (Hermle, Z 206 A, Wehingen, Germany). The obtained biomass after centrifugation was filtered, then washed with deionized water to remove any impurities, and centrifuged again. The obtained biomass after centrifugation was extracted with a ten times higher volume of methanol at room temperature for 24 h. The extraction was repeated three times with the same biomass. The obtained methanol extracts were combined after filtration through glass fiber filters (0.6 µm) and evaporated using a rotary evaporator (RV 10 basic, IKA, Staufen, Germany) under reduced pressure at 40 °C [1,31]. After solvent removal, crude biomass extract was obtained and stored in refrigeration at 4 °C until further use for the determination of its hemolytic and genotoxic properties. The extraction procedure was conducted in the Biochemical Laboratory of the Department of Chemistry at the Faculty of Science, University of Kragujevac.

### 2.3. Cyanotoxin Detection

The detection of cyanotoxins was performed according to the WHO protocol [1,31] at the accredited laboratory of the Institute of Public Health of Serbia, by ‘Dr. Milan Jovanović Batut’.

#### 2.3.1. Chemicals and Reagents

Microcystin LR (MC-LR), microcystin DMLR (MC-DMLR), microcystin DMRR (MC-DMRR), microcystin RR (MC-RR), and microcystin YR (MC-YR), were supplied by Enzo Life Sciences (Lausen, Switzerland). Anatoxin-a fumarate (ATX) and cylindrospermopsin (CYN) were supplied by Chem Cruz (Santa Cruz Biotechnology, Dalas, TX, USA). All the standards were >95% purity (HPLC). Methanol (MeOH) of MS grade (99.99%) and dichloromethane (DCM) of analytical reagent grade (99.9%) were obtained from Fisher Scientific (Leics, UK) and acetonitrile MS grade (99.99%) was obtained from Carlo Erba (Val de Reuil Cedex, France). High purity water was produced on-site using Barnstead GenPure (Thermo Scientific, Waltham, MA, USA). Sodium hydroxide (NaOH) 2 M used for sample pH adjustment, was prepared from pellets (purity 98%), purchased from Sigma Aldrich (Steinheim, Germany). Formic acid (FA) (99.9%) was prepared from Carlo Erba (Val de Reuil Cedex, France).

#### 2.3.2. Solid Phase Extraction

SPE was carried out using an assembly of two cartridges, Plexa HLB (500 mg, Agilent, Santa Clara, CA, USA) and HyperSep Hypercarb PGC (porous graphitic carbon, 500 mg, Thermo Scientific, Altrincham, UK). The SPE was carried out using a 12-port SPE vacuum manifold with Altech and a diaphragm vacuum pump (Barnant Co., Barrington, IL, USA). The optimization of SPE was performed by Zervou et al. [32]. The optimized SPE procedure was as follows: 4 mL of methanol was added to 400 mL of sample. The initial sample pH was adjusted to 11 with the addition of NaOH 2 M. Plexa HLB and HyperSep PGC cartridges were connected in series (top Oasis HLB, bottom PGC) and conditioned sequentially with 6 mL of DCM, 6 mL of methanol, and 6 mL water (pH11). The samples were passed through the SPE assembly. The cartridges were dried for 15 min (air under vacuum). The sequence of the two cartridges in the SPE assembly was then reversed (top PGC, bottom Plexa HLB) and the analytes were eluted with a mixture of 10 mL DCM:MeOH (40:60, *v*/*v*), containing 0.5% FA. The extract was dried under a gentle stream of nitrogen. The residue was re-dissolved with 400 µL MeOH: H2O (5:95, *v*/*v*) and sonicated in a water bath for 5 min (Bandelin Sonorex, Berlin, Germany). The final extract was then transferred to an autosampler glass vial and analyzed by LC-ESI–MS.

#### 2.3.3. Liquid Chromatography–Mass Spectrometry

LC–MS was carried out on an ISQ EM mass spectrometer (Thermo Scientific, Waltham, MA, USA), equipped with an electrospray ionization (ESI) source. Separation of the target analytes was achieved with a Dionex Ultimate 3000 LC system, equipped with a Dionex Ultimate AS autosampler (Thermo Scientific, Waltham, MA, USA), and Dionex Ultimate 3000 Column compartment (Thermo Scientific, Waltham, MA, USA). Chromeleon 7.2.10 (Thermo Scientific, Waltham, MA, USA) was used to control chromatographic, mass spectrometric parameters and for data acquisition. For chromatographic separation, reversed phase chromatographic Acclaim Polar Advantage II, 4.6 × 150 mm, 3 µm (Thermo Scientific, Waltham, MA, USA) column was used.

Optimum chromatographic separation was achieved using a gradient elution program with (A) water and (B) can, both containing 0.1% FA. The gradient program started at 20% A (held for 3 min), increasing to 70% A for 22 min (held for 3 min). An equilibration time of 8 min was needed after each sample run. The flow rate was set at 0.3 mL min^−1^, the column was thermostated at 30 °C, and the injection volume was 10 µL. The retention times (tR) for each identified cyanotoxin are presented in Table 1.

The ionization of the compounds was performed with an ESI probe in positive mode. Detection was carried out via single ion monitoring (SIM) using molecular ions. The optimized ionization conditions were set as follows: sheath gas pressure of 35.8 psi, auxiliary gas pressure of 4.0 psi, sweep gas pressure of 0.0 psi, and an ion transfer tube temperature of 275 °C, while the vaporizer temperature was set to 350 °C. The source voltage was set to 3.5 kV.

#### 2.3.4. Method Validation

After the selection of the optimum conditions for LC–MS, the method was validated. The method specificity, the linearity, the range of measurement, the trueness, the precision, and the limits of detection/quantification were assessed. Standard solutions at five different concentrations (20, 50, 100, 200, 500 ng mL^−1^) of the 7 toxins were analyzed in triplicates, so as to evaluate the measurement and calibration range. For the assessment of specificity, blank samples were analyzed. The trueness was evaluated via the analysis of the water samples spiked with the analytes in triplicate at the concentrations level of 75, 150, and 450 ng mL^−1^. Experiments for precision were performed in six replicates each day for two different days (n = 12) at concentration of 100 ng mL^−1^. The limits of detection (LODs) were estimated according to the International Conference on Harmonization Guideline [33], based on repeated measurements (n = 6) of standard solutions (5 ng mL^−1^) by the signal-to-noise ratio formula LOD = 3.3 S/N.

### 2.4. Measurement of DNA Damage

#### 2.4.1. Blood Sampling

Human peripheral blood was collected via venipuncture from three healthy, nonsmoking donors (4 mL per donor) of mean age 22.67 ± 0.58 years (age range of 22–23 years) who had not been exposed to known mutagens in the last six months. The donors were informed about the aims and methodology of the study and each donor signed informed consent according to the guidelines of the Declaration of Helsinki.

#### 2.4.2. Alkaline Comet Assay

The DNA damage was evaluated using the alkaline version of the single-cell gel electrophoresis/comet assay according to the procedure described by Collins et al. [34] with alterations suggested by Tubić Vukajlović et al. [35]. All the experiments were conducted in the dark to avoid possible photo-induced DNA damage. The isolation of the human peripheral blood lymphocytes (PBLs) was performed using Histopague-1077 (Sigma Aldrich, St. Louis, Missouri, USA). The lymphocytes were centrifuged (Sigma 2-16KL, UK) for 15 min at 550× *g* and washed twice in RPMI medium (Sigma Aldrich, St. Louis, Missouri, USA) by centrifugation for 10 min at 500× *g*. Then, they were resuspended in phosphate-buffered saline (PBS) solution and incubated for half an hour at 37 °C with five different concentrations (10, 25, 50, 75, and 100 µg mL^−1^) of the cyanobacteria extract dissolved in PBS. Negative (untreated cells) and positive control (H_2_O_2_, 10 µg mL^−1^) were parallelly included, too. After the incubation, 100 µL of cell suspension was mixed with 100 µL of 1% agarose low melting point (dissolved in PBS, in a final concentration of 0.01 g/mL) (AppliChem GmbH, Darmstadt, Germany) and spread into pre-gelatinized slides (per two drops of 90 μL). When the agarose solidified (for few seconds on ice), the slides were immersed in freshly prepared lysing solution (2.5 M NaCl, 100 mM EDTA (Centrohem, Stara Pazova, Serbia), 10 mM Tris (Sigma Aldrich, St. Louis, Missouri, USA), 1% Triton X-100 (Fisher Scietific, Geel, Belgium), and 10% dimethyl sulfoxide (Centrohem, Stara Pazova, Serbia, pH 10) for 2 h and were immersed in an electrophoresis buffer (10 M NaOH, 200 mM EDTA (Centrohem, Stara Pazova, Serbia, pH > 13) to allow alkaline denaturation. The electrophoresis (Bio-Rad, PowerPac Basic, Hercules, CA, USA) was performed at 4 °C, 25 V (0.7 V/cm), and 300 mA for 30 min. The slides were then washed three times for 15 min with neutralizing Tris-HCl buffer (0.4 M, pH 7.5, Sigma Aldrich, St. Louis, Missouri, USA) and rinsed in distilled water. The alkaline comet assay was conducted in the Genetics Laboratory of the Department of Biology and Ecology at the Faculty of Science, University of Kragujevac.

#### 2.4.3. Cell Analysis

The slides were stained with 50 µL ethidium bromide (20 µg mL^−1^, Sigma Aldrich, USA) and studied using a fluorescent microscope (Nikon E50i) at 400x magnification. Based on the degree of DNA damage, the cells (size and intensity of the comet tails) were visually classified into five classes (0–4): category 0—no damage; category 1—low damage; category 2—medium damage; category 3—high damage; and category 4—total damage. A total of 100 cells per donor was analyzed (50 cells from each of the two replicate gels). The percentage of DNA (%DNA) damage was calculated according to the following formula:

%DNA damage = Class1 + Class2 + Class3 + Class4

The Genetic Damage Index (GDI) was obtained by summing up the number of cells in each class multiplied by the class number, giving a rating between 0 and 4, and dividing by the total number of analyzed cells, using the following formula [36]:

$$GDI=\frac{Class1+2\times Class2+3\times Class3+4\times Class4}{Class0+Class1+Class2+Class3+Class4}$$

### 2.5. Hemolytic Activity

The possible hemolytic activity of the methanol extract of the cyanobacteria biomass was tested using erythrocyte cells (RBCs) obtained from three healthy human donors. Blood samples for the isolation of erythrocytes obtained from healthy individuals, according to the protocol described in Section 2.4.1, were collected in sterile containers with ethylenediaminetetraacetic acid (EDTA, Sigma Aldrich, St. Louis, Missouri, USA) as an anticoagulant. Samples from all the donors were separately centrifugated at 1000× *g* for 10 min and obtained precipitated erythrocytes were washed three times using phosphate-buffer saline (PBS, pH 7.4) and then centrifuged under the same conditions. Cyanobacteria biomass extract in concentrations of 200, 100, 75, 50, 25, and 10 µg mL^−1^ were prepared in PBS, and 1 mL of these solutions was mixed with 1 mL of 5% erythrocytes suspension in PBS. As a negative control, only PBS was used instead of the extract, while a 1% solution of sodium dodecyl sulfate (SDS Sigma Aldrich, Darmstadt, Germany) was used for the positive control (considered to cause 100% hemolysis). After incubation at 37 °C for 1 h, the samples were centrifuged at 250× *g* for 15 min. Then, the absorbance of the supernatant was measured spectrophotometrically (Halo DB-20S, Dynamica GmbH, Dietikon, Switzerland) at 540 nm [37]. All the experiments were performed in triplicate for erythrocytes from each donor. The percentage of hemolyzed erythrocytes was calculated using the following equation:

$$\%hemolysis=\frac{{(A}_{s}-A_{0})-A_{k}}{A_{SDS}-A_{k}}\times 100$$

where *A_s_* is the absorbance of samples with extract, *A*_0_ is the absorbance of the corresponding concentration of extract in PBS, *A_SDS_* is the positive control with SDS solution, and *A_k_* is the negative control (only RBCs in PBS).

### 2.6. Statistical Analyses

Statistical analysis was performed using the SPSS (version 20) software package and the results were expressed as mean ± standard deviation (SD). A one-way analysis of variance (ANOVA), with Tukey’s post hoc test, was used for the comparisons of the treatments with the negative control. The relationship between the tested extract concentrations and both % DNA damage and GDI values was determined using the Pearson correlation coefficient. Differences with *p* < 0.05 and *p* < 0.0005 were considered statistically significant.

## 3. Results

**Figure 1:**
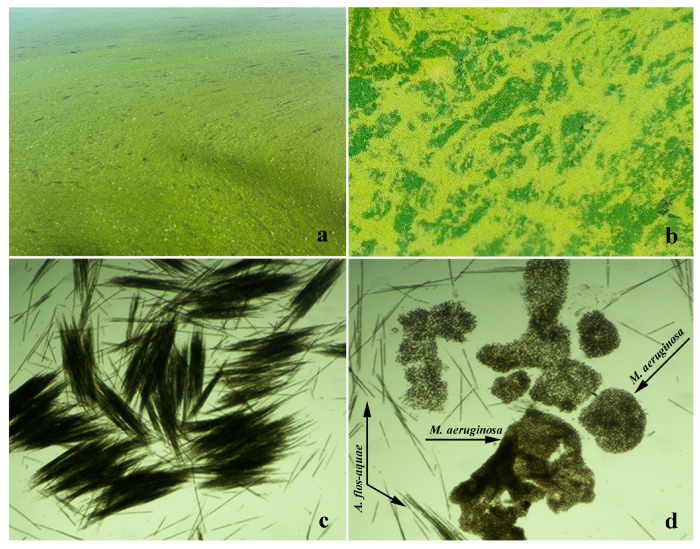
The blooming of *A. flos-aquae* and *M. aeruginosa* in the Gruža reservoir (October 2023): (**a**,**b**) cyanobacterial blooms; (**c**) microscope images of *A. flos-aquae*; and (**d**) microscope images of *M. aeruginosa* (arrows) and *A. flos-aquae* (arrows).

During the sampling period at the Gruža reservoir in Serbia, the blooming species *Aphanizomenon flos-aquae* and *Microcystis aeruginosa* were highly abundant (Figure 1). The highest number of cyanobacterial cells recorded was 4,656,450 cells mL^−1^, with the dominant species being *A. flos-aquae* (3,105,120 cells mL^−1^). This species, along with *M. aeruginosa* (1,480,130 cells mL^−1^), was the most abundant in the reservoir. Together, these two species accounted for 98.5% of the total phytoplankton in the Gruža reservoir. Due to their absolute dominance, sufficient material was available for extract preparation.

The analysis of the extract revealed the presence of the following cyanotoxins, expressed in µg g^−1^ of biomass: anatoxin-a—3.56 µg g^−1^, cylindrospermopsin—6.86 µg g^−1^, microcystin LR—0.87 µg g^−1^, and microcystin RR—2.47 µg g^−1^.

**Figure 2:**
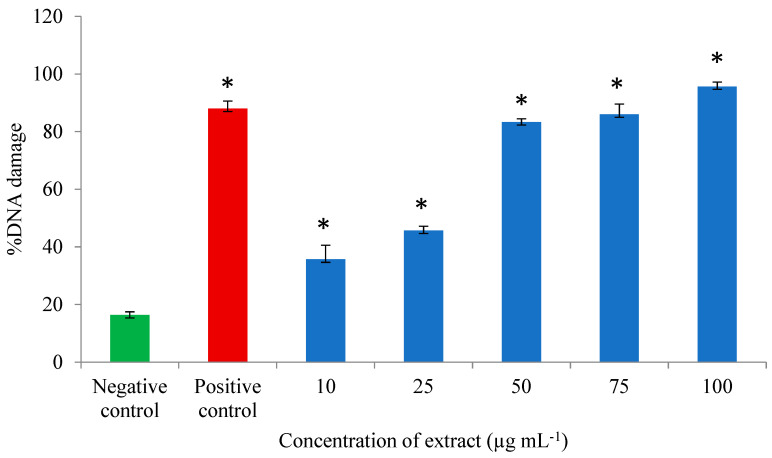
Level of %DNA damage in PBLs of healthy control persons after treatment with methanolic extract of cyanobacteria biomass *A. flos-aquae* and *M. aeruginosa.* * Statistically significant increase in %DNA damage compared to negative control (ANOVA *p* < 0.0005).

**Figure 3:**
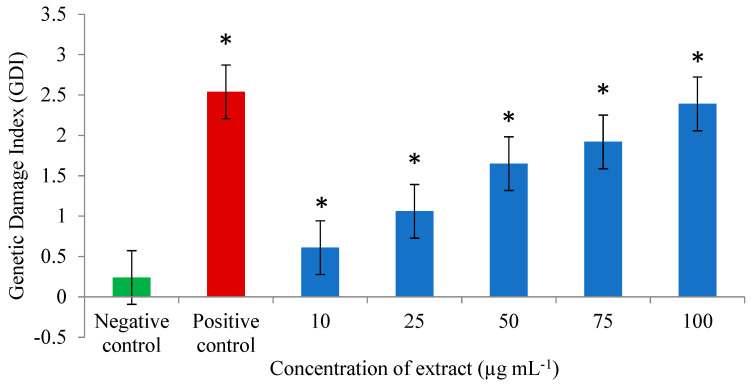
Genetic Damage Index in PBLs of healthy control persons after treatment with methanolic extract of cyanobacteria biomass *A. flos-aquae* and *M. aeruginosa.* * Statistically significant increase GDI compared to negative control (ANOVA *p* < 0.0005).

The results of the in vitro genotoxicity potential of the *A. flos-aquae* and *M. aeruginosa* extract are shown in Figure 2 and Figure 3. The percentage of DNA damage varied from 15 to 17/100 analyzed cells, while the GDI varied from 0.22 to 0.27 in untreated PBLs. At the lowest concentration (10 µg mL^−1^), the %DNA damage varied from 30 to 39, and the GDI varied from 0.61 to 0.63, and increased proportionally with the extract concentration so that in the highest concentration (100 µg mL^−1^), the range of %DNA damage was 94 to 97 and the GDI was 2.31 to 2.44.

All the tested concentrations of the methanolic extract significantly increased (*p* < 0.0005) the average values of the %DNA damage (from 35.67 ± 4.93 to 95.67 ± 1.53) and the GDI (from 0.61 ± 0.02 to 2.39 ± 0.07) in comparison to the negative control (16.33 ± 1.15 for %DNA damage and 0.24 ± 0.03 for GDI). The genotoxic effect of the extract was concentration-dependent (Pearson correlation coefficient: r = 0.968, *p* = 0.000 for %DNA damage and r = 0.996 for GDI; *p* = 0.000). Also, with the increase in extract concentrations, the percentage of undamaged cells decreased about 15 times compared to the lowest concentration (1.44 vs. 21.44%), and about 20 times compared to the negative control (1.44 vs. 27.89%).

**Figure 4:**
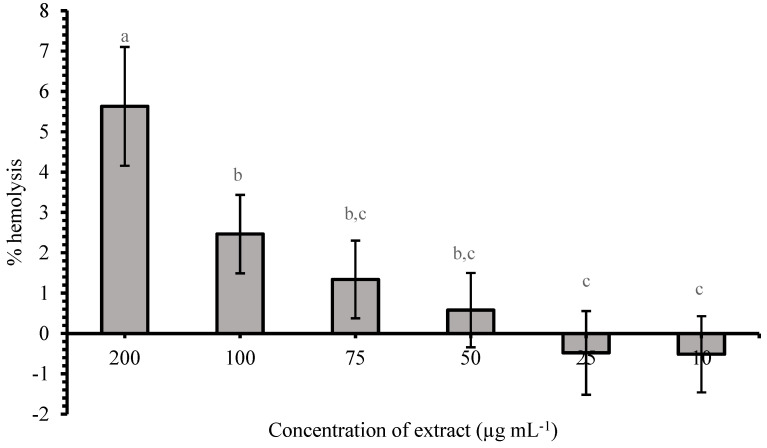
Percentage of hemolysis induced by different concentrations of cyanobacteria biomass methanolic extract. Results are presented as a mean of the tree replication of the experiment performed separately on erythrocytes obtained from 3 healthy human donors ± standard deviation (SD). Values with different letters are significantly different (*p* < 0.05), as determined using Tukey’s post hoc test.

Before the manifestation of nephrotoxicity and hepatotoxicity, cyanobacteria toxins may also be found in the bloodstream where they interact with blood cells, including erythrocyte cells. Therefore, it is important to examine their hemolytic activity. The obtained results of the hemolytic activity of cyanobacteria methanol extract using erythrocytes from human blood samples are presented in Figure 4. In this study, SDS as a positive control, used in a concentration of 1%, is considered to cause the complete hemolysis of erythrocytes (100%). The percentages of hemolysis caused by cyanobacteria biomass extract were calculated relative to this value obtained for SDS.

According to the obtained results, the methanol extract of the cyanobacteria biomass caused dose dependent hemolysis, but in lower percentages (Figure 4). It can be said that in higher concentrations, 100 and 200 µg mL^−1^, the extract that contains different cyanobacteria toxins may cause 2 to 5% of erythrocytes hemolysis, approximately, while in concentrations between 50 and 100 µg mL^−1^ may cause only 1 to 2% of hemolysis. The percentages of hemolysis obtained with concentrations of 100, 75, and 50 µg mL^−1^ are not significantly different from each other (*p* > 0.05), but are significantly lower (*p* < 0.05) compared to the percentage of hemolysis at a concentration of 200 µg mL^−1^. The mean percentage of red blood cell hemolysis at the highest cyanobacteria extract concentration (200 µg mL^−1^) was 5.63%. With a decrease in the applied concentration of the extract, the percentage of hemolysis decreased proportionally, so that the percentage of hemolysis at the lowest applied concentrations (10 and 25 µg mL^−1^) was lower than the negative control. The results also indicate that although the concentrations of 75 and 50 µg mL^−1^ caused hemolysis levels ranging from 1.5% to 0.5%, no statistically significant differences (*p* > 0.05) were observed when compared to the treatments with concentrations of 25 and 10 µg mL^−1^.

## 4. Discussion

Our study demonstrated that the mixed blooming biomass of *Aphanizomenon flos-aquae* and *Microcystis aeruginosa* species, collected directly from the natural environment without laboratory cultivation, was toxic, although it remains unclear which specific species were responsible for the observed toxicity. On the other hand, it should be noted that one-time water analyses conducted during periods of intense cyanobacterial blooms did not detect the presence of microcystins [38]. Given the alarming levels of cyanotoxins detected in the cyanobacterial biomass in the Gruža Reservoir for the first time, the potential health risks posed by these toxins cannot be ignored, particularly in drinking water, irrigation, and recreational activities, all of which are common uses of this water source.

As mentioned, most studies have focused on the presence and concentration of toxins in water rather than their direct biological effects [22]. Specifically, the genotoxic effects of cyanotoxins from the biomass of *A. flos-aquae* and *M. aeruginosa* on human cells are not well-documented. Đorđević et al. [39] conducted in vivo studies on laboratory rats to monitor the genotoxic potential of cylindrospermopsin; although some research has investigated the effects of *M. aeruginosa*, data are still limited [40], and the specific impact of *A. flos-aquae* on DNA damage in healthy human cells has not been thoroughly studied. This gap in the literature prompted us to assess the genotoxic potential of these cyanobacterial extracts on human peripheral blood lymphocytes in vitro.

Our results showed that the methanolic extract obtained from the biomass of cyanobacteria (*A. flos-aquae* and *M. aeruginosa*) was genotoxic in all the tested concentrations. Namely, the level of DNA damage significantly increased in the treatments from the lowest to the highest concentration compared to the untreated cells. These results are in agreement with the results of Žegura et al. [40] who examined the induction of DNA strand-breaks in cultured human PBLs with concentrations 0.1, 1, and 10 µg mL^−1^, from microcystins from *M. aeruginosa,* using the comet assay. They concluded that microcystins induced a dose-dependent increase in DNA damage in PBLs. Applying the same assay, Mankiewicz-Boczek et al. [41] came to the same conclusion when examining the genotoxic effect of microcystins from the same cyanobacteria at a concentration of 0.08 to 2 µg mL^−1^ in PBLs. It is known that *A. flos-aquae* is a harmful form of cyanobacteria that produces the toxicants saxitoxin and cylindrospermopsin [42]. The genotoxicity study of Žegura et al. [43] indicates that cylindrospermopsin significantly increases the level of DNA damage even in low concentrations (0.05, 0.1 and 0.5 µg mL^−1^) in cultured PBLs. Žegura et al. [40,43] concluded that microcystins and cylindrospermopsin should be considered genotoxic agents and that PBLs can be targets of this agent-induced genotoxicity.

Considering the well-known risks for health issues caused by cyanobacteria toxins, such as liver and kidney damage, gastrointestinal symptoms (e.g., nausea, vomiting, diarrhea), and, in certain situations, neurological problems [44,45], there is not much data on their effect on blood cells. An earlier study by Grabow et al. [46] showed that human, mouse, rat, sheep, and musk duck erythrocytes were lysed within minutes by the cyanobacteria *M. aeruginosa* toxin, with the hemolysis dependent on the temperature [47]. Interestingly, the study conducted by Benedetti et al. [47] showed that phycocyanin extract from the cyanobacteria *A. flos-aquae* had a protective role against red blood cells, oxidative hemolysis, and lipid peroxidation induced by the aqueous peroxyl radical generator [2,2′-Azobis (2-amidinopropane) dihydrochloride, AAPH] [47]. Previous studies showed that some cyanobacteria toxins have an influence on the structure of the erythrocyte membrane. The study that tracked the effects of microcystin LR on human erythrocytes at doses ranging from 1 to 1000 nM over time found that these toxins caused hemolysis in erythrocytes, changed oxyhemoglobin into methemoglobin, and reduced membrane fluidity [48,49]. In addition, a long-term study conducted by Sukenik et al. [50] and a short-term study conducted by Reisner et al. [51] showed that exposure of red blood cells to cylindrospermopsin leads to a disruption in the normal structure of erythrocytes, whereby it acquires a spiculated form known as spiculated red cells with a few projections of varying size and surface distribution [50,51]. However, there are many studies about the toxic effects of anatoxin-a, but no literature data that confirms its hemolytic activity. Many studies confirm its neurotoxic effect; specific targets for anatoxin-a are nicotinic acetylcholine receptors in the central nervous system, leading to the disruption of nerve impulses and potentially causing paralysis or respiratory failure [52]. The low hemolytic potential of the examined cyanobacteria biomass extract may be related to the low concentration of microcystin LR in the extract, which is reliably known to have strong hemolytic potential.

## 5. Conclusions

This study highlights the substantial genotoxic effects of cyanotoxins produced by the biomass of *Aphanizomenon flos-aquae* and *Microcystis aeruginosa* on human blood cells. The high concentrations of these cyanobacteria in the Gruža reservoir, coupled with the presence of potent cyanotoxins such as anatoxin-a, cylindrospermopsin, and microcystins, underscore the serious health risks associated with exposure to contaminated water sources. The observed dose-dependent increase in DNA damage and hemolysis emphasizes the need for the rigorous monitoring and management of water quality, particularly in reservoirs used for drinking water. These findings contribute to our understanding of the cellular mechanisms by which cyanotoxins exert their harmful effects and underscore the necessity for stricter regulations and proactive interventions to mitigate exposure to contaminated water sources, especially those supplying drinking water, to safeguard public health.

## Figures and Tables

**Table 1:** Retention time and monitored ion of cyanotoxins.

Cyanotoxin	Retention Time (min)	Single Ion Monitoring (m z^−1^)
Anatoxin-a	4.06	166 [M + H]^+^
Cylindrospermopsin	5.50	416 [M + H]^+^
Microcystin DMRR	14.55	512 [M + 2H]^+2^
Microcystin RR	14.95	520 [M + 2H]^+2^
Microcystin DMLR	18.70	982 [M + H]^+^
Microcystin YR	19.04	1046 [M + H]^+^
Microcystin LR	19.25	996 [M + H]^+^

## Data Availability

The original contributions presented in the study are included in the article, further inquiries can be directed to the corresponding author.

## References

[1] Chorus I., Welker M. (2021). Toxic Cyanobacteria in Water.

[2] Rickert B., Chorus I., Schmoll O. (2016). Protecting Surface Water for Health: Identifying, Assessing and Managing Drinking-Water Quality Risks in Surface-Water Catchments.

[3] Ho J.C., Michalak A.M., Pahlevan N. (2019). Widespread global increase in intense lake phytoplankton blooms since the 1980s. Nature.

[4] Bănăduc D., Curtean-Bănăduc A., Barinova S., Lozano V.L., Afanasyev S., Leite T., Branco P., Gomez Isaza D.F., Geist J., Tegos A. (2024). Multi-Interacting Natural and Anthropogenic Stressors on Freshwater Ecosystems: Their Current Status and Future Prospects for 21st Century. Water.

[5] Huisman J., Codd G.A., Paerl H.W., Ibelings B.W., Verspagen J.M., Visser P.M. (2018). Cyanobacterial blooms. Nat. Rev. Microbiol..

[6] Zepernick B.N., Wilhelm S.W., Bullerjahn G.S., Paerl H.W. (2022). Climate change and the aquatic continuum: A cyanobacterial comeback story. Environ. Microbiol. Rep..

[7] Simić S., Đorđević N., Milošević D.J. (2017). The relationship between the dominance of Cyanobacteria species and environmental variables in different seasons and extreme precipitation. Fund. Appl. Limnol..

[8] Sinha E., Michalak A.M., Balaji V. (2017). Eutrophication will increase during the 21st century as a result of precipitation changes. Scince.

[9] Wagner C., Adrian R. (2009). Cyanobacteria dominance: Quantifying the effects of climate change. Limnol. Oceanogr..

[10] Meriluoto J., Spoof L., Codd G.A. (2017). EU-COST Handbook of Cyanobacterial Monitoring and Cyanotoxin Analysis.

[11] Simić S., Đorđević N., Tokodi N., Drobac Backović D., Marinović Z., Simić V., Simić S., Pešić V. (2023). Eutrophication of Fishing Waters and the Influence of Cyanobacterial Occurrence and Blooming on Fish Resources: Case Studies in Serbia. Ecological Sustainability of Fish Resources of Inland Waters of the Western Balkans.

[12] Svirčev Z., Lalić D., Bojadžija Savić G., Tokodi N., Drobac Backović D., Chen L., Meriluoto J., Codd G.A. (2019). Global geographical and historical overview of cyanotoxin distribution and cyano-bacterial poisonings. Arch. Toxicol..

[13] Prüss-Ustün A., Wolf J., Bartram J., Clasen T., Cumming O., Freeman M.C., Gordon B., Hunter P.R., Medlicott K., Johnston R. (2019). Burden of disease from inadequate water, sanitation and hygiene for selected adverse health outcomes: An updated analysis with a focus on low-and middle-income countries. Int. J. Hyg. Environ. Health.

[14] Wood R. (2016). Acute animal and human poisonings from cyanotoxin exposure—A review of the literature. Environ. Int..

[15] Frigg R., Hartmann S., Zalta E.N. (2020). Models in Science. The Stanfod Encyclopedia of Philosophy.

[16] Kiviranta J., Sivonen K., Niemelä S.I., Huovinen K. (1991). Detection of Toxicity of *Cyanobacteria* by *Artemia salina* Bioassay. Environ. Toxicol. Water Qual..

[17] Maršalek B., Bláha L. (2004). Comparison of 17 Biotests for Detection of Cyanobacterial Toxicity. Environ. Toxicol..

[18] Berry J.P., Gibbs P.D.L., Schmale M.C., Saker M.L. (2009). Toxicity of *Cylindrospermopsin*, and Other Apparent Metabolites from *Cylindrospermopsis raciborskii* and *Aphanizomenon ovalisporum*, to the Zebrafish (*Danio rerio*) Embryo. Toxicon.

[19] Davidović P. (2023). Toxicity of Selected Strains of Cyanobacteria in In Vivo and In Vitro Tests. Ph.D. Thesis.

[20] Nizan S., Dimentman C., Shilo M. (1986). Acute Toxic Effects of the Cyanobacterium *Microcystis aeruginosa* on *Daphnia magna*. Lim-nol. Oceanogr..

[21] Dao T.S., Do-Hong L.C., Wiegand C. (2010). Chronic Effects of Cyanobacterial Toxins on *Daphnia magna* and Their Offspring. Toxicon.

[22] Davidović P., Blagojević D., Meriluoto J., Simeunović J., Svirčev Z. (2023). Biotests in Cyanobacterial Toxicity Assessment—Efficient Enough or Not?. Biology.

[23] Yilmaz O., Patinote A., Nguyen T., Bobe J. (2018). Multiple Vitellogenins in Zebrafish (*Danio rerio*): Quantitative Inventory of Genes, Transcripts and Proteins, and Relation to Egg Quality. Fish. Physiol. Biochem..

[24] Sazdova I., Keremidarska-Markova M., Chichova M., Uzunov B., Nikolaev G., Mladenov M., Schubert R., Stoyneva-Gärtner M., Gagov H.S. (2022). Review of Cyanotoxicity Studies Based on Cell Cultures. J. Toxicol..

[25] Ćomić L., Ostojić A. (2005). Akumulaciono Jezero Gruža (The Gruža Reservoir—In Serbian).

[26] Arsenijević M. (2020). Algal Diversity and Ecological Potential of the Gruža Reservoir During the Autumn of 2019. Master’s Thesis.

[27] (2016). Water Quality—Guidance on Quantitative and Qualitative Sampling of Phytoplankton from Inland Waters.

[28] Utermöhl H. (1958). Zur Vervollkomnung der quantitativen Phytoplankton-Methodik. Int. Ver. Theor. Angew. Limnol. Mitteilungen.

[29] Komárek J., Anagnostidis K., Büdel B., Gärtner G., Krienitz L., Schagerl M. (1998). Cyanoprokaryota 1. Teil: Chroococcales. Süsswasserflora von Mitteleuropa, Bd. 19/1.

[30] Komárek J., Büdel B., Gärtner G., Krienitz L., Schagerl M. (2013). Cyanoprokaryota 3. Teil: Heterocystous Genera. Süßwasserflora von Mitteleuropa, Bd. 19/3.

[31] Harada K., Kondo F., Lawton L., Chorus I., Bartram J. (1999). Laboratory analysis of cyanotoxins, chapter 13. Toxic Cyanobacteria in Water: A Guide to Their Public Health Consequences, Monitoring and Management (WHO).

[32] Zervou S.-K., Christophoridis C., Kaloudis T., Triantis T.M., Hiskia A. (2017). New SPE-LC-MS/MS method for simultaneous determination of multi-class cyanobacterial and algal toxins. J. Hazard. Mater..

[33] ICH Harmonised Tripartite Guideline (2022). Validation of Analytical Procedures: Text and Methodology.

[34] Collins A., Møller P., Gajski G., Vodenková S., Abdulwahed A., Anderson D., Eyluel Bankoglu E., Bonassi S., Boutet-Robinet E., Brunborg G. (2023). Measuring DNA modifications with the comet assay: A compendium of protocols. Nat. Protoc..

[35] Tubić Vukajlović J., Simić I., Smiljanić Z., Grujičić D., Milošević-Djordjević O. (2023). Genome instability in peripheral blood lymphocytes of patients with heart failure and reduced ejection fraction. Mutagenesis.

[36] Pitarque M., Vaglenov A., Nosko M., Hirvonen A., Norppa H., Creus A., Marcos R. (1999). Evaluation of DNA damage by the comet assay in shoe workers exposed to toluene and other organic solvents. Mutat. Res./Genet. Toxicol. Environ. Mutagen..

[37] Srećković N.Z., Nedić Z.P., Monti D.M., D’Elia L., Dimitrijević S.B., Mihailović N.R., Katanić Stanković J.S., Mihailović V.B. (2023). Biosynthesis of Silver Nanoparticles Using *Salvia pratensis* L. Aerial Part and Root Extracts: Bioactivity, Biocompatibility, and Catalytic Potential. Molecules.

[38] Public Utility Company “Water and Sewerage” Kragujevac. https://jkpvik-kg.com/.

[39] Đorđević N.B., Matić S.L., Simić S.B., Stanić S.M., Mihailović V.B., Stanković N.M., Stanković V.D., Ćirić A.R. (2017). Impact of the toxicity of *Cylindrospermopsis raciborskii* (Woloszynska) Seenayya & Subba Raju on laboratory rats in vivo. Environ. Sci. Pollut. Res. Int..

[40] Žegura B., Gajski G., Štraser A., Garaj-Vrhovac V., Filipič M. (2011). Microcystin-LR induced DNA damage in human peripheral blood lymphocytes. Mutat. Res./Genet. Toxicol. Environ. Mutagen..

[41] Mankiewicz-Boczek J., Palus J., Gagała I., Izydorczyk K., Jurczak T., Dziubałtowska E., Stepnik M., Arkusz J., Komorowska M., Skowron A. (2011). Effects of microcystins-containing cyanobacteria from a temperate ecosystem on human lymphocytes culture and their potential for adverse human health effects. Harmful Algae.

[42] Seo Y., Yoon Y., Lee M., Jang M., Kim T., Kim Y., Yoo H.Y., Min J., Lee T. (2023). Rapid electrochemical biosensor composed of DNA probe/iridium nanoparticle bilayer for *Aphanizomenon flos-aquae* detection in fresh water. Colloid Surf. B.

[43] Žegura B., Gajski G., Štraser A., Garaj-Vrhovac V. (2011). Cylindrospermopsin induced DNA damage and alteration in the expression of genes involved in the response to DNA damage, apoptosis and oxidative stress. Toxicon.

[44] Bláha L., Babica P., Maršálek B. (2009). Toxins produced in cyanobacterial water blooms—Toxicity and risks. Interdiscip. Toxicol..

[45] Qiu T., Xie P., Li L., Guo L., Zhang D., Zhou Q. (2012). Nephrotoxic effects from chronic toxic cyanobacterial blooms in fishes with different trophic levels in a large Chinese lake. Environ. Toxicol. Pharmacol..

[46] Grabow W.O., Du Randt W.C., Prozesky O.W., Scott W.E. (1982). *Microcystis aeruginosa* toxin: Cell culture toxicity, hemolysis, and mutagenicity assays. Appl. Environ. Microbiol..

[47] Benedetti S., Benvenuti F., Pagliarani S., Francogli S., Scoglio S., Canestrari F. (2004). Antioxidant properties of a novel phycocyanin extract from the blue-green alga *Aphanizomenon flos-aquae*. Life Sci..

[48] Sicińska P., Bukowska B., Michałowicz J., Duda W. (2006). Damage of cell membrane and antioxidative system in human erythrocytes incubated with microcystin-LR in vitro. Toxicon.

[49] Lone Y., Bhide M., Koiri R.K. (2016). Microcystin-LR Induced Immunotoxicity in Mammals. J. Toxicol..

[50] Sukenik A., Reisner M., Carmeli S., Werman M. (2006). Oral toxicity of the cyanobacterial toxin cylindrospermopsin in mice: Long-term exposure to low doses. Environ. Toxicol. Int. J..

[51] Reisner M., Carmeli S., Werman M., Sukenik A. (2004). The cyanobacterial toxin cylindrospermopsin inhibits pyrimidine nucleotide synthesis and alters cholesterol distribution in mice. Toxicol. Sci..

[52] Matsunaga S., Moore R.E., Niemczura W.P., Wayne W., Carmichael W.W. (1989). Anatoxin-a(s), a potent anticholinesterase from *Anabaena flos-aquae*. J. Am. Chem. Soc..

